# Serum From Melioidosis Survivors Diminished Intracellular *Burkholderia pseudomallei* Growth in Macrophages: A Brief Research Report

**DOI:** 10.3389/fcimb.2020.00442

**Published:** 2020-08-26

**Authors:** Panjaporn Chaichana, Barbara Kronsteiner, Patpong Rongkard, Prapit Teparrukkul, Direk Limmathurotsakul, Narisara Chantratita, Nicholas P. J. Day, Helen A. Fletcher, Susanna J. Dunachie

**Affiliations:** ^1^Mahidol-Oxford Tropical Medicine Research Unit, Faculty of Tropical Medicine, Mahidol University, Bangkok, Thailand; ^2^Peter Medawar Building for Pathogen Research, University of Oxford, Oxford, United Kingdom; ^3^Center for Tropical Medicine and Global Health, University of Oxford, Oxford, United Kingdom; ^4^Medical Department, Sunpasitthiprasong Hospital, Ubon Ratchathani, Thailand; ^5^Department of Tropical Hygiene, Mahidol University, Bangkok, Thailand; ^6^Department of Microbiology and Immunology, Faculty of Tropical Medicine, Mahidol University, Bangkok, Thailand; ^7^Department of Immunology and Infection, London School of Hygiene and Tropical Medicine, London, United Kingdom

**Keywords:** melioidosis, functional antibodies, opsonization, phagocytosis, *Burkholderia pseudomallei*, growth inhibition, bacteria

## Abstract

Melioidosis is a neglected tropical disease with high mortality rate. It is caused by the Gram-negative, CDC category B select agent *Burkholderia pseudomallei* (*B. ps*) that is intrinsically resistant to first-line antibiotics. An antibody-based vaccine is likely to be the most effective control measure. Previous studies have demonstrated significant mechanistic roles of antibodies in protection against death in animal models, but data from human melioidosis is scarce. Herein, we used *in-vitro* antibody-dependent cellular phagocytosis and growth inhibition assays to assess the mechanism of protective antibodies in patients with acute melioidosis. We found that serum from patients who survived the disease enable more live *B. ps* to be engulfed by THP-1 derived macrophages (median 1.7 × 10^3^ CFU/ml, IQR 1.1 × 10^3^-2.5 × 10^3^ CFU/ml) than serum from patients who did not survive (median 1.2 × 10^3^ CFU/ml, IQR 0.7 × 10^3^-1.8 × 10^3^, *p* = 0.02). In addition, the intracellular growth rate of *B. ps* pre-opsonized with serum from survivors (median 7.89, IQR 5.58–10.85) was diminished when compared with those with serum from non-survivors (median 10.88, IQR 5.42–14.88, *p* = 0.04). However, the difference of intracellular bacterial growth rate failed to reach statistical significance when using purified IgG antibodies (*p* = 0.09). These results provide new insights into a mechanistic role of serum in protection against death in human melioidosis for antibody-based vaccine development.

## Introduction

Melioidosis is well-recognized as a major cause of fatal community-acquired septicemia in northeast Thailand and north Australia (Chaowagul et al., 1989; Currie et al., 2000). The case fatality rate can exceed 40% in endemic regions. Melioidosis is now known to be endemic in at least 45 countries across tropical areas, and 89,000 global deaths per annum are estimated (Limmathurotsakul et al., 2016). The causative agent of melioidosis is the facultative intracellular Gram-negative bacillus *Burkholderia pseudomallei* (*B. ps*), which is intrinsically resistant to commonly used antibiotics. Prolonged antibiotic regimens are required to control the infection. Highly effective vaccines against other polysaccharide-encapsulated bacteria including *Streptococcus pneumoniae, Neisseria meningitides*, and *Haemophilus influenzae* type b rely on antibody-mediated protection (Snape et al., 2008; Pollard et al., 2009; Wahid et al., 2014), hence antibody-based vaccines are one of the most promising approaches to control *B. ps* infection.

Various studies have reported an association between protection against melioidosis and humoral immune responses in animal models (Scott et al., 2013; Silva et al., 2013; Burtnick et al., 2018; Khakhum et al., 2019); however, mechanistic roles of the protective antibodies against melioidosis in humans have not been well-elucidated.

The role of antibody quantity in protection against human melioidosis is uncertain. High antibody titers against whole cell *B. ps* antigens were not associated with protection in melioidosis patients (Norazah et al., 1996; Chaichana et al., 2018). Besides, high background titers in individuals living in endemic regions makes interpretation not straight-forward (Ho et al., 1997; Chaichana et al., 2018). However, a recent research has reported a correlation between survival and high levels of antibodies specific to lipopolysaccharide (LPS) II (Charuchaimontri et al., 1999) and Hemolysin co-regulated protein 1 (Hcp1) (Pumpuang et al., 2019).

Antibody titers alone do not always account for protective immunity. Therefore, this study aimed to adopt *in vitro*-based approaches to measure the functional roles of antibodies associated with protection against death in acute human melioidosis.

## Materials and Methods

### Ethics Statement

The study was approved by the ethics committees of the Faculty of Tropical Medicine, Mahidol University (Submission number TMEC 12-014); of Sunpasithiprasong Hospital, Ubon Ratchathani (reference 018/2555); and the Oxford Tropical Research Ethics Committee (reference 64-11). The study was conducted according to the principles of the Declaration of Helsinki (2008), and the International Conference on Harmonization (ICH) Good Clinical Practice (GCP) guidelines. Written informed consent was obtained for all patients enrolled in the study.

### Serum Sample Collection From Acute Melioidosis Patients

Two hundred adult in-patients with acute melioidosis aged > 18 years old at Sunpasithiprasong Hospital, Ubon Ratchathani, Thailand were enrolled, at a median of 5 days (interquartile range (IQR) 3–6, range 2–13) after admission, as described previously (Jenjaroen et al., 2015). Blood, sputum, throat, endotracheal, bronchoalveolar lavage, pus, or urine were collected on the day of enrollment for bacteria isolation. Melioidosis was defined as isolation of *B. ps* from any clinical sample. Serum and plasma were separated from blood after centrifugation of clotted and heparinized blood, respectively, before aliquoted and stored at −80°C until used. The 28-days case fatality rate in this cohort was 26%. Healthy control subjects with no diabetes or other medical problem were recruited from the blood donation clinic at Sunpasitthiprasong Hospital. Age range of healthy subjects were from 28 to 73 years old (median 44, IQR 41–52). 23/50 of healthy subjects who have indirect hemagglutination assay (IHA) titer was <1:10 were selected as seronegative controls for melioidosis. 136/200 patients in the melioidosis cohort had sufficient stored serum for assays (*n* = 94 for survivors and *n* = 42 for non-survivors).

### Cell Lines and Bacteria Cultures

The human monocyte (THP-1) cell line was cultured in complete medium (10%FCS RPMI-1640) at 37°C with 5% CO_2_ for 2 days before used in the assays. To generate THP-1 derived macrophage-like cells, THP-1 cells were incubated with 1 μM Phorbol 12-myristate 13-acetate (PMA, Sigma, Germany) for 3 days. The cells were then washed three times to remove PMA, and rested in complete medium for 3 days before use.

*B. ps* strain K96243 (from frozen stock) was spread onto Columbia agar and incubated at 37°C. The overnight culture from a single colony was grown in tryptone-soy broth (TSB) and incubated overnight at 37°C with shaking at 200 rpm. Then, the culture was diluted with TSB to achieve an OD_600_ of 0.05, and further incubated at 37°C shaking until it reached log-phase (OD_600_ of 0.6). The bacteria were washed 3 times with PBS and kept at −80°C in aliquots until used.

### IgG Purification

Total IgG antibodies were individually purified from 100 μl of the plasma of each melioidosis patient in the cohort via negative selection using Melon™ Gel IgG Spin Purification Kit (Thermo Scientific, MA) according to the manufacturer's protocol. After purification, 82/136 patients in the cohort had sufficient concentration (≥ 3 mg/ml) of the antibodies for assays (*n* = 55 for survivors and *n* = 27 for non-survivors).

### Antibody-Dependent Cellular Phagocytosis (ADCP) Assay

Frozen log-phase *B. ps* was thawed, washed and incubated with fluorescein isothiocyanate (FITC) dye for 1 h at 37°C in the dark. The FITC-labeled *B. ps* was then washed thoroughly with PBS 3 times. Live *B. ps*, unlabeled or FITC-labeled, were incubated with heat-inactivated (56°C, 30 min) patient serum (10% v/v), purified IgG (0.5 mg/ml) or RPMI-1640 (control) at 37°C for 30 min. The *B. ps* and antibody or control mixtures were then added to THP-1 cells at a ratio of 5 colony forming units (CFUs)/cell. After a 15-min incubation at 37°C, the co-culture mixtures were immediately chilled on ice to stop phagocytosis. The cells were washed twice with PBS, and incubated with chilled trypan blue (Sigma) at 4°C for 15 min to quench the FITC signal of cell surface-bound *B. ps* (Busetto et al., 2004). The cells were then washed twice with PBS and incubated with Cytofix^TM^ fixation buffer (BD Bioscience, CA, USA) at 4°C for 15 min. After washing the cells were resuspended in cold MACSQuant Running Buffer (Miltenyi Biotec, Germany), and analyzed by flow cytometry using the MACSQuant® Analyzer 10 (Miltenyi Biotec). Results are expressed as Phagocytic index, as described below:

(1) $$\text{Phagocytic index}=\frac{\%\text{ infected cell in the presence of antibodies}}{\%\text{ infected cell in the absence of antibodies}}$$

### Growth Inhibition Assay

The unlabeled *B. ps*-antibody mixtures as described above were added to THP-1 derived-macrophages a ratio of 0.01 CFU/cell and further incubated for 1 h. In case of purified IgG, the unlabeled *B. ps* was incubated with purified antibodies at the concentration of 0.5 mg/ml for 30 min at 37°C before transferred to the cells. The extracellular bacteria were killed by a 2-h incubation in 500 μg/ml of kanamycin. At selected time points, the infected cells were washed and lysed with 1% saponin (Sigma) in PBS. The intracellular bacteria were diluted in sterile water and plated onto Columbia agar. The CFUs were counted and expressed as CFU/ml. Bacterial survival is expressed as growth rate of bacteria at 7 h compared with 3 h-post infection.

(2) $$\text{Growth rate }=\frac{\left(\frac{\text{CFU}}{\text{ml}}\text{at 7 h post infection})\text{ − }(\frac{\text{CFU}}{\text{ml}}\text{at 3 h post infection}\right)}{\frac{\text{CFU}}{\text{ml}}\text{ at 3 h post infection}}$$

### Statistical Analysis

Non-normally distributed continuous data were reported as median and interquartile range (IQR). The significance of differences between two groups was analyzed by Mann-Whitney U-test in GraphPad Prism 7 for Windows (GraphPad Software, San Diego, CA, USA).

## Results

### Survivors of Melioidosis Have Elevated ADCP Activity Compared to Non-survivors

**Figure 1:**
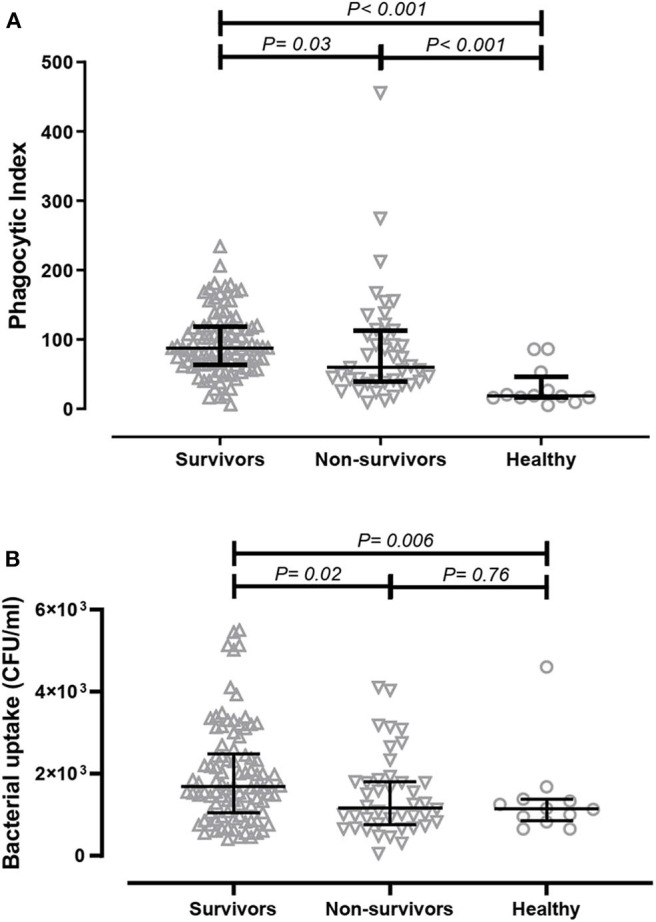
Comparison of *in-vitro* antibody-dependent cellular phagocytosis (ADCP) activity of serum derived from acute melioidosis patients who survived the disease (upward open triangles), acute melioidosis fatal cases (downward open triangles) and healthy endemic controls (open circle). **(A)** The ADCP activity in human monocyte THP-1 cells were determined by flow cytometry and expressed as phagocytic index (described in Material and Methods). **(B)** The ADCP activity of antibodies was also determined in THP-1-derived macrophages using the colony count method to detect live bacteria inside infected cells. The statistical test used for comparison is the Kruskal-Wallis one-way ANOVA for more than two groups, and Mann-Whitney *U* for two groups.

ADCP is important for the clearance of bacterial infection; thus, we initially determined the opsonophagocytic activities of antibodies derived from patients in a monocyte cell line using flow cytometry. Serum samples were collected from acute melioidosis patients at a median of 5 days after admission, and antibodies against *B. ps* antigens in the serum were measured by ELISA. The median absorbance value (OD_450_) of IgG against heat-killed whole cell *B. ps* in survivors and non-survivors was comparable (Chaichana et al., submitted manuscript). The phagocytic index of serum from healthy controls containing undetectable level of *B. ps*-specific antibodies was significantly lower than that from melioidosis patients (both survivors and non-survivors). We observed a significantly greater phagocytic index in serum samples derived from melioidosis patients who survived the disease (median 87.53, IQR 63.71–118.80) compared to those who died (median 60.39, IQR 39–113.30, *p* = 0.03) and healthy controls (median 18.60, IQR 16.09–46.29, *p* < 0.001, Figure 1A).

We subsequently measured the amount of live *B. ps* inside of THP-1-derived macrophages upon ADCP using a conventional colony count method. The bacterial uptake when using serum from survivors (median 1.6 × 10^3^ CFU/ml, IQR 1.1 × 10^3^-2.5 × 10^3^ CFU/ml) was significantly higher compared to using serum from non-survivors (median 1.2 × 10^3^, IQR 0.7 × 10^3^-1.8 × 10^3^, *p* = 0.02) and healthy controls (median 1.1 × 10^3^, IQR 0.9 × 10^3^- 1.4 × 10^3^, *p* = 0.03, Figure 1B).

### Intracellular Bacterial Survival Is Enhanced in The Presence of Serum From Fatal Cases Compared to Survivors

**Figure 2:**
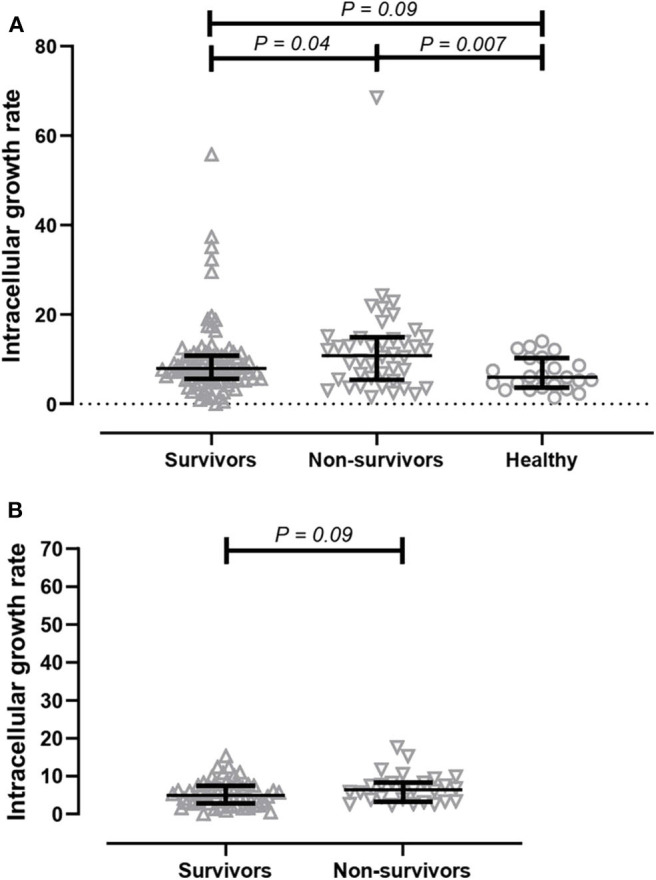
Comparison of intracellular bacterial growth inhibition assay (GIA) in THP-1 derived macrophages. **(A)** GIA using serum from acute melioidosis patients who survived the disease (upward open triangles), acute melioidosis fatal cases (downward open triangles), and healthy endemic controls (open circle). **(B)** GIA using purified IgG antibodies from acute melioidosis patients who survived the disease (upward open triangles) and acute melioidosis fatal cases (downward open triangles). The result is expressed as fold change in CFU/ml at 7 h post-infection compared to 3 h post-infection. The statistical test used for comparison is the Kruskal-Wallis one-way ANOVA for more than two groups, and Mann-Whitney *U* for two groups.

We next determined the effect of serum on intracellular bacterial survival in THP-1-derived macrophages. We measured the growth rate of bacteria after a 3-h incubation period. We found that intracellular growth rates of *B. ps* inside human macrophages were significantly higher in the presence of serum from non-survivors (median 10.88, IQR 5.42–14.88) when compared to serum from survivors (median 7.89, IQR 5.58–10.85, *p* = 0.04) and healthy donors (median 6.04, IQR 3.22–7.87, *p* = 0.01, Figure 2A).

Next, we performed an intracellular growth inhibition assay using total IgG individually purified from patients to remove other plasma proteins and components that may affect the result. There is a borderline evidence showing that the intracellular growth rate of *B. ps* inside human macrophages was higher in the presence of purified IgG from non-survivors (median 6.36, IQR 3.36–8.29) when compared to IgG from survivors (median 4.96, IQR 2.80–7.5, *p* = 0.09, Figure 2B).

## Discussion

In this study, we assessed the functional role of antibodies in protection against death from acute melioidosis. We found that serum from acute melioidosis patients who survived the disease showed significantly enhanced uptake of live *B. ps* to be engulfed by THP-1-derived macrophages compared to those from non-survivors. Moreover, the serum from survivors also diminished intracellular growth of *B. ps* in macrophages after phagocytosis. These results reveal a significant contribution of humoral immune responses in protection against death in human melioidosis.

Although statistical significance was not achieved, purified IgG individually purified from survivors seems to enhance intracellular bacterial growth inhibition when compared to IgG from non-survivors. This result relates to the total IgG level in serum. Borderline *P*-value of 0.09 in this study probably due to an insufficient number of samples, hence replicate trial with increased number of samples should be conducted to confirm this finding. In addition, the purified IgG from survivors and non-survivors seems to enhance intracellular growth inhibition activity compared to serum samples. This result was probably due to the higher quantity of antibodies in the assay system when using purified IgG. The concentration of IgG in serum of general adult population varies at different age ranging from 400 to 2,200 mg/deciliter blood (Gonzalez-Quintela et al., 2008). We need to measure the concentration of total IgG in serum of melioidosis patients to confirm this explanation.

In separate study, we found that the serum level of total IgG and IgM was not significantly different between survivors and non-survivors (Chaichana et al., submitted manuscript). However, we did find that the level of IgG2 subclass was significantly higher in patients who survived the disease compared to those who did not. Previous studies demonstrated that the IgG2 level was associated with protective immunity (Zarei and Redwan, 2019) and defense against recurrence of capsulated bacterial infections (Kralickova et al., 2017; Yamazaki et al., 2019). Follow up work includes a comparison between the intracellular bacterial growth rate of live *B. ps* pre-opsonized with purified IgG2 antibodies derived from survivors and non-survivors.

Both ADCP activity and intracellular growth inhibition in this study are complement-independent processes as we heat-inactivated the serum before use. Therefore, the different outcomes between serum and purified IgG may be due to other components in the serum. C-type lectins, which are heat-resistant plasma opsonins, are also implicated in phagocytosis of pathogens via C-type lectin-like receptors on myeloid cells (Kang et al., 2005; Chiffoleau, 2018). Further analysis of C-type lectins on opsono-phagocytosis and intracellular growth inhibition is required.

The ADCP activity of serum from healthy controls with undetectable anti-*B. ps* antibodies used to determine the background level of the assays (Chaichana et al., 2018). We found that FITC-labeled *B. ps* pre-opsonized with serum from fatal cases showed a significantly higher phagocytic index than serum from healthy controls. Unexpectedly, the phagocytic activity was comparable between fatal cases and healthy controls when measuring the number of live bacteria by conventional colony count. Serum from fatal cases probably results in more FITC-labeled dead bacteria to be engulfed by macrophages than those from healthy controls; however further analysis is required to prove this effect.

Serum from patients who survived melioidosis increased *B. ps* uptake and diminished the intracellular growth in THP-1-derived macrophages, but the macrophages could not clear the bacteria. This result was similar to a previous study in a mouse model in which pre-opsonization of *B. ps* with *B. ps* LPS- and capsule-specific IgG increased uptake, but did not promote clearance in primary murine macrophages (Mulye et al., 2014). THP-1 cells are derived from tumor cells that are not equivalent to those of macrophages obtained from circulating monocytes in terms of their phenotypic and molecular properties (Kohro et al., 2004; Chaichana et al., 2017). In addition, the THP-1-derived macrophages used in our study were in a naïve, not-activated, M0-like state which is characterized by slower production of reactive oxygen species (ROS) upon phagocytic stimulation when compared to an IFN-γ-activated M1-like state (Mendoza-Coronel and Ortega, 2017). Therefore, we need to further evaluate the intracellular bacterial growth inhibition of serum or antibodies comparing primary human and THP-1-derived macrophages activated with LPS or IFN-γ to become pro-inflammatory classical M1 macrophages that have high capacity for bacterial killing.

We found that antibodies from some non-survivors have a higher phagocytic index value than survivors, but these antibodies have low or moderate potential to inhibit intracellular bacterial growth in macrophages. This result implies that the antibodies may use distinct mechanism between bacterial uptake and intracellular killing. Moreover, it is also possible that some patients who do not survive in spite of a strong functional antibody response may suffer from other contributing factors in the disease pathway, such as high inoculating pathogen burden, late presentation, poorer T cell responses, or undetected underlying comorbidities.

To our knowledge, this is the first report demonstrating that intracellular bacterial growth inhibition in macrophages is one of the important mechanistic roles of serum in protection against death in human melioidosis. Replication in independent melioidosis cohorts is required to confirm these antibody functions. The antigenic targets of these functional antibodies also need to be identified for development of effective antibody-based vaccines. The *in-vitro* ADCP and intracellular growth inhibition assays from this study offer a powerful tool to assess the efficacy of vaccine candidates against melioidosis in animal models and vaccine trials.

## Data Availability Statement

The raw data supporting the conclusions of this article will be made available by the authors, without undue reservation.

## Ethics Statement

The studies involving human participants were reviewed and approved by the ethics committees of the Faculty of Tropical Medicine, Mahidol University (Submission number TMEC 12-014); of Sunpasithiprasong Hospital, Ubon Ratchathani (reference 018/2555); and the Oxford Tropical Research Ethics Committee (reference 64-11). The patients/participants provided their written informed consent to participate in this study.

## Author Contributions

PC performed the experiments, analyzed the data, wrote the manuscript. PT conducted the clinical study. BK, PR, DL, NC, ND, HF, and SD conceived the research, designed experiments, interpreted data, and corrected the manuscript. All authors read, commented on and agreed the content of the manuscript.

## Conflict of Interest

The authors declare that the research was conducted in the absence of any commercial or financial relationships that could be construed as a potential conflict of interest.
